# A reconfigurable sandwich structure switchable DNA-based metamaterial

**DOI:** 10.1038/s41598-020-74214-6

**Published:** 2020-10-15

**Authors:** Min Zhong

**Affiliations:** Hezhou University, Hezhou, 542899 China

**Keywords:** Biophysics, Materials science, Optics and photonics, Physics

## Abstract

In this paper, a tunable DNA-based metamaterial is designed and simulated in 170–340 THz range. This metamaterial can be transformed from an ON mode with a low resistance state of the DNA strip to its OFF mode with a high resistance state. Three Structures with containing different combinations metal layers are designed and simulated. Structure 1 with *Ag*/*DNA*/*Ag* and *Au*/*DNA*/*Au* strategies achieves field enhancement factors (FEF) 2.18 and 2.07, respectively. Structure 2 (*Au*/*DNA*/*Dirac*, *Dirac*/*DNA*/*Au*, *Ag*/*DNA*/*Dirac*, or *Dirac*/*DNA*/*Ag*) achieves the FEF values 14.11, 10.70, 13.75, or 9.62, respectively, while the FEF value of Structure 3 with *Dirac*/*DNA*/*Dirac* reaches 59.8. The FEF value of Structure 3 can be modulated from 59.8 to 91.96 as Fermi energy increasing from 0 to 60 meV. Moreover, the FEF value is also enhanced through increasing the magnetic field strength. The Structure 3 exhibits convertibility and sustainable modulation lines between two opposing patterns. The proposed structure reveals a switchable feature based on the resistance characteristics of DNA strips, which can be revealed as an ON/OFF switch sensor. Moreover, the switching performance of Structures 3 and 2 is significantly higher than Structure 1. Therefore, Structures 3 and 2 can be set to be an optical memristor or optical gate.

## Introduction

Metamaterial shows unique properties based on the inclusion of artificially designed microstructures: lensing, negative refraction index, and cloaking^1–4^. Metamaterials are proposed and applied in many fields, such as absorber, sensor, and so on^5–13^. With the development and expansion of research, tunable metamaterial has become the focus of researchers. Based on tunable metamaterial, continuous modulation of electromagnetic wave signals in a certain frequency band can be realized, for example, liquid crystal, VO_2_ and GST^14–16^. Dirac semimetal is a research hotspot in recent years, which is also regarded as “3-D graphene”. Dirac semimetals contain many types, such as Na3Bi, AlCuFe quasicrystals, and Cd_3_As_2_^17–19^. A metallic response can be achieved by the Dirac semimetal layer when the frequency lower is than Fermi energy, while a dielectric response is obtained when the frequency is higher than Fermi energy^20^. Therefore, tunable properties can be found in the Dirac semimetal layer. For example, the carrier mobility of Dirac semimetal layer is up to $9\, \times \,10^{6} \,{\text{cm}}^{2} /{\text{V}}/{\text{s}}$ at temperature point 5 k, which is higher than that of graphene^21,22^. This phenomenon means that the properties of the Dirac semimetal can be modulated by changing temperature. The permittivity functions of Dirac semimetal layer can be adjusted through changing the Fermi energy^23,24^. Therefore, the Dirac semimetal is an efficient, continuously controllable, tunable metamaterial. To date, Dirac semimetal is rarely used in metamaterial devices with two opposite modes. On the other hand, more and more convertible materials are used to develop metamaterial devices, such as graphene, or indium-tin oxide (ITO)^25,26^. Recently, biological material (DNA) has attracted the attention of researchers and been applied to the development of convertible devices^27,28^. The switchable characteristic can be achieved based on the ON (low resistance) and OFF (high resistance) modes of DNA materials^29^. However, both modes (ON, OFF) of these DNA-based metamaterials can’t be continuously modulated. This is because these metamaterials do not contain material elements that can be continuously modulated. Combining the tunable metamaterial and convertible DNA-strip to achieve a newly metamaterial device is important and interesting.

In this paper, a DNA-based metamaterial waveguide is designed and simulated in 170-340THz range. Three structural schemes are proposed and validated: Structure 1(*Au*/*DNA*/*Au* or *Ag*/*DNA*/*Ag*), Structure 2 (*Au*/*DNA*/*Dirac*, *Dirac*/*DNA*/*Au*, *Ag*/*DNA*/*Dirac*, or *Dirac*/*DNA*/*Ag*), Structure 3 (*Dirac*/*DNA*/*Dirac*). These structural schemes achieve different FEF values. Structure 3 with two Dirac semimetal layers has a significantly higher FEF than that of the Structures 1 and 2. Moreover, the Structure 3 can be modulated continuously through varying the Fermi energy or the magnetic field strength.

## Structural design and model introduction

**Figure 1:**
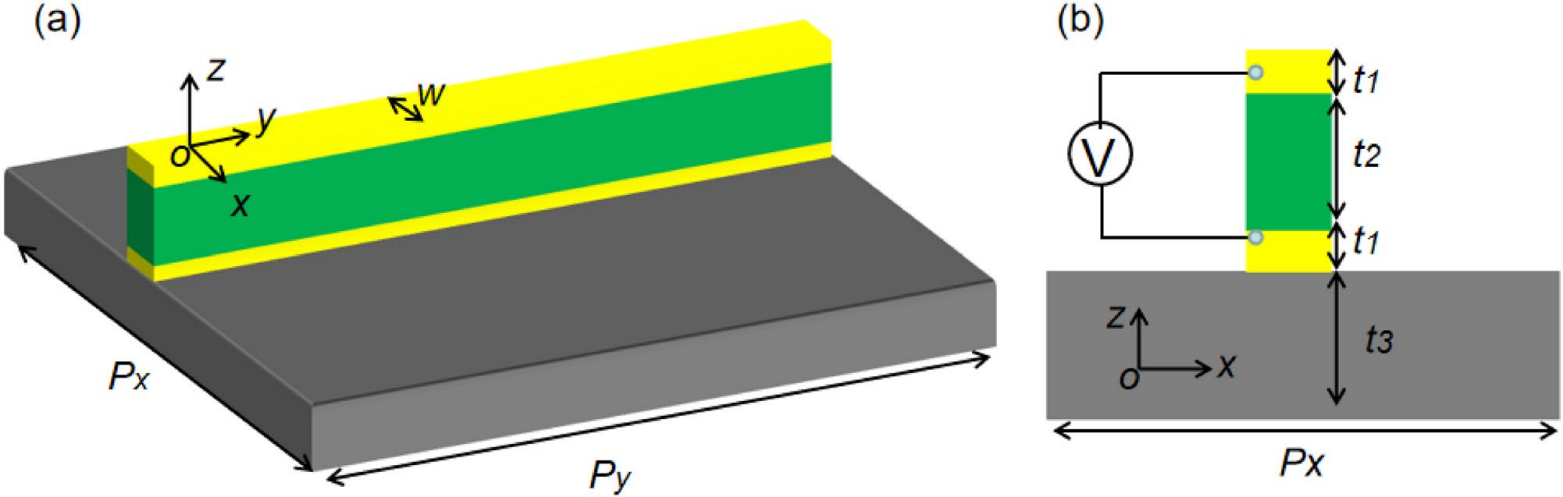
(**a**) Three-dimensional image of the proposed structure. (**b**) Cross section of the proposed structure. The yellow parts are metal layers. The green part is the DNA strip. The gray part is the substrate.

The proposed DNA-based metamaterial can be found in Fig. 1, which contains four layers: Two metal layers (these metal layers can be selected as gold, silver, or Dirac semimetal) are separated by a DNA strip. The thickness of the air layer above and below the unit is: 1000 nm and 500 nm, respectively.

### Simulations

Simulations are obtained by HFSS. The step size of the simulation frequency is 0.01 THz. The upper and lower layers are 6 and 3 microns thick, respectively. Perfectly matched layers are applied to the structural unit. The bottom layer is a silicon dioxide layer, and the dielectric constant of this silicon dioxide layer is 2.105^30^. The electromagnetic wave enters along the longitudinal direction of the waveguide. Ideal electric or magnetic boundaries are used^31^.

### Model

In this paper, metal (gold or silver) layers are given as follows^32^:

1 $$\varepsilon (\omega ) = 1 - \frac{{\omega_{p}^{2} }}{{\omega^{2} - i\omega \gamma_{D} }}$$

In the Eq. (1) above, $\varepsilon (\omega )$ is the dielectric constant of metal layers, $\omega$ is the simulated frequency, $\omega_{p}^{{}} { = 1}{\text{.37}} \times {10}^{16} \,{\text{s}}^{ - 1}$ is set to be the plasma frequency, $\gamma_{D} { = 9}{\text{.0}} \times {10}^{13} \,{\text{s}}^{ - 1}$ is set to be the collision frequency. The Dirac semimetal layer is achieved as follows^33^:

2 $${\text{Re}} \sigma (\Omega ) = \frac{{gk_{F} e^{2} }}{24\pi \hbar }\Omega G\left( {\frac{\Omega }{2}} \right)$$

3 $${\text{Im}} \sigma (\Omega ) = \frac{{gk_{F} e^{2} }}{{24\pi^{2} \hbar }}\left\{ {\frac{4}{\Omega }\left[ {1 + \frac{{\pi^{2} }}{3}\left( {\frac{T}{{E_{F} }}} \right)^{2} } \right] + 8\Omega \int\limits_{0}^{{\varepsilon_{c} }} {\left[ {\frac{G(\varepsilon ) - G(\Omega - 2)}{{\Omega^{2} - 4\varepsilon^{2} }}} \right]\varepsilon d\varepsilon } } \right\}$$

In the Eqs. (2, 3) above, the $G(E)$ is achieved as $G(E) = {\text{n(}} - {\text{E)}} - {\text{n(E)}}$, and the ${\text{n(E)}}$ is defined as Fermi distribution function, the ${\text{E}}_{c} { = }3$ is defined as the cutoff energy, Fermi level is $E_{F}$, Fermi momentum can be achieved as $k_{F} = E_{F} /\hbar v_{F}$, Fermi velocity is achieved as $v_{F} = 10^{6} \,{\text{m}}/{\text{s}}$. Moreover,$\varepsilon = E/E_{F}$, $\varepsilon_{c} { = }E_{c} /E_{F}$, and $\Omega = \hbar \omega /E_{F}$. $g$ is set to be the degeneracy factor. The permittivity of Dirac semimetal layer is given as follows^34^:

4 $$\varepsilon = \varepsilon_{b} + {\text{i}}\sigma {/}\omega \varepsilon_{0}$$

Here, the $\varepsilon_{0}$ is defined as the permittivity of vacuum. The $\varepsilon_{b}$ is defined as the background dielectric. It is well know that the organic or protein material is always shown switching characteristic due to the variable resistivity. For example, the conductivity of the organic material, such as the DNA strip (or other protein strip), can be changed through modulating the electric field. As is known to all, genetic information is storage in the DNA, which shows many resonance properties, such as self-assembling, nano-size effect, and mechanical rigidity. Therefore, the DNA strip can be applied in nanometer-scale electronics^35^. For example, DNA strip is applied in designed and fabricated memory devices^36,37^. Unfortunately, DNA strip is rarely used in metamaterial. Therefore, it makes sense to design and develop tunable metamaterial using DNA strip with dual mode properties. In this paper, the thickness, width, and length of the DNA strip are 100 nm, 80 nm, and 100 nm, as shown in Fig. 1. In this paper, the simulated DNA strip is set to be “ON” or “OFF” modes for the low resistance of 5 × 10^9 ^$\Omega$ or the high resistance of 5 × 10^10 ^$\Omega$^38,39^. The detailed structural parameters of the proposed DNA-based metamaterial are shown in Table 1.

**Table 1:** Geometric parameters.

Parameter	Px	Py	w	t_1_	t_2_	t_3_
Value (nm)	400	100	80	10	100	110

## Results and discussion

### Structure 1 with *Au*/*DNA*/*Au* or *Ag*/*DNA*/*Ag*

**Figure 2:**
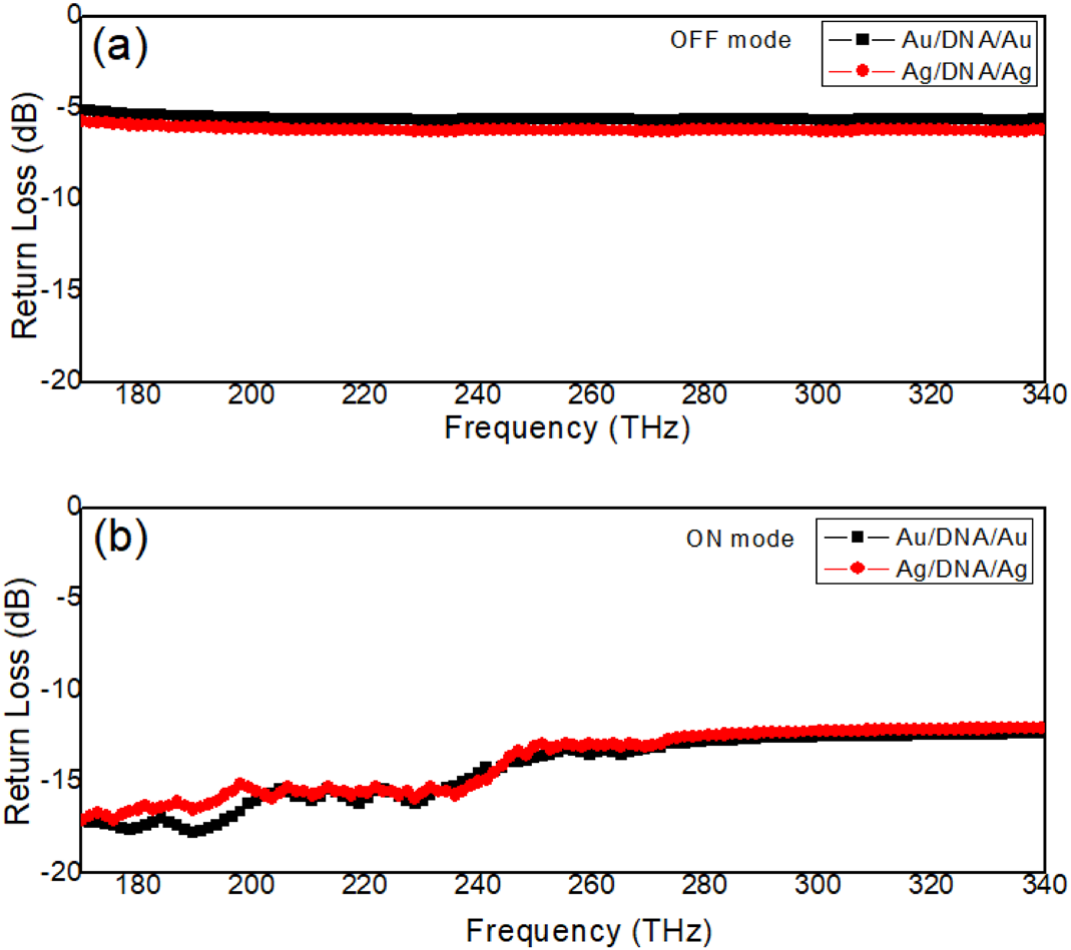
(**a**) The return loss value of the *Au*/*DNA*/*Au* and *Ag*/*DNA*/*Ag* metamaterial waveguide for the OFF mode. (**b**) The return loss value of the *Au*/*DNA*/*Au* and *Ag*/*DNA*/*Ag* metamaterial waveguide for the ON mode.

**Figure 3:**
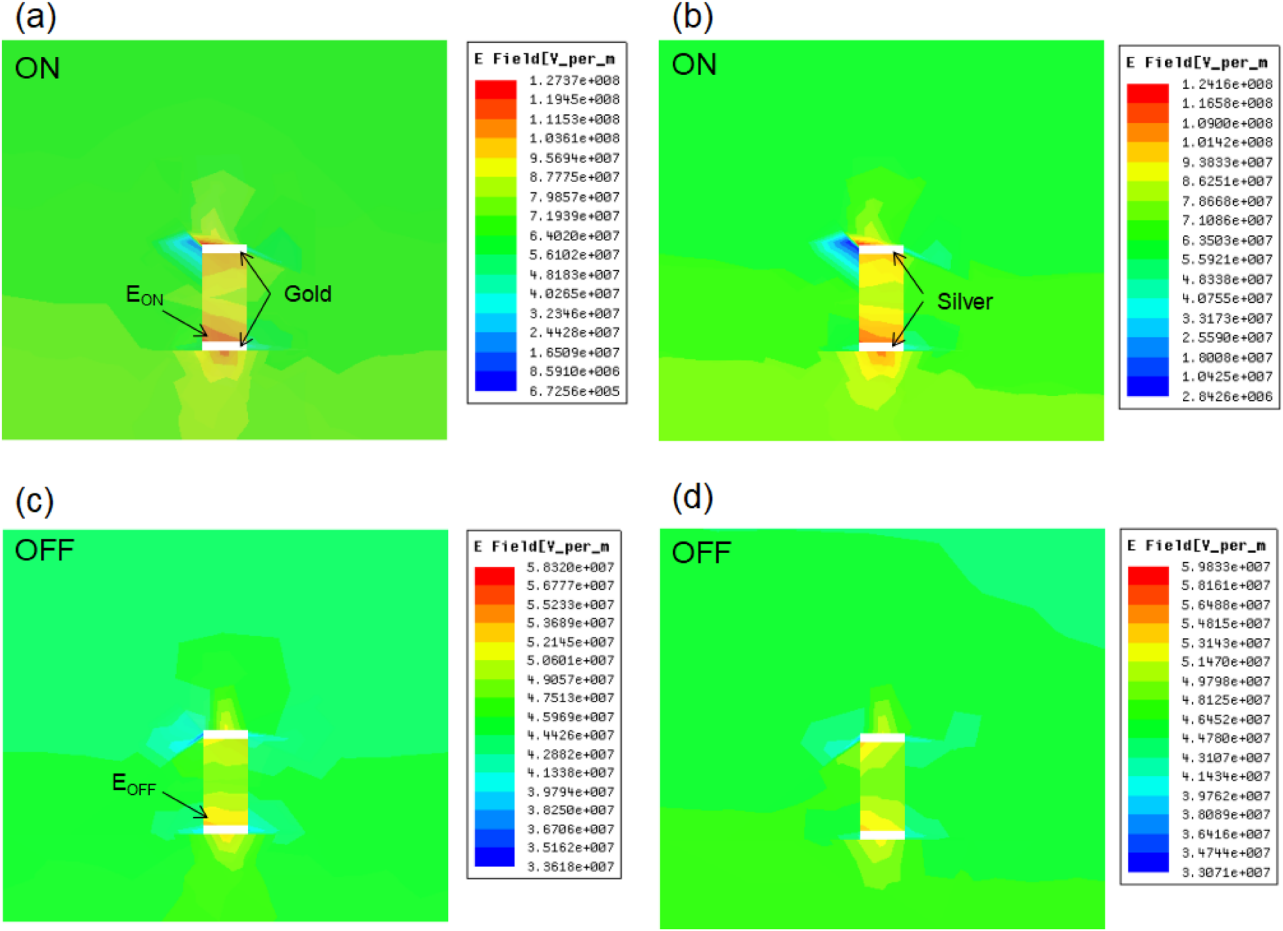
(**a**) The electric field intensity at the end of the waveguide for the ON mode of the Structure 1 with *Au*/*DNA*/*Au*. (**b**) The electric field intensity at the end of the waveguide for ON mode of the Structure 1 with *Ag*/*DNA*/*Ag*. (**c**) The electric field intensity at the end of the waveguide for OFF mode of the Structure 1 with *Au*/*DNA*/*Au*. (**d**) The electric field intensity at the end of the waveguide for OFF mode of the Structure 1 with *Ag*/*DNA*/*Ag*.

The proposed metamaterial structure contains a DNA strip coating with two gold layers on both sides, the conductivity properties of this DNA strip play an important role on the transmission and electric field distributions. Figure 2a shows the return loss value of the Structure 1 with *Au*/*DNA*/*Au* in 170THz to 340THz. When the DNA strip shows the ON mode (resistance of 5 × 10^9 ^$\Omega$)^38,39^, the return loss value is about -14.5 dB, while the DNA strip shows the OFF mode (resistance of 5 × 10^10 ^$\Omega$), the return loss value increases to − 5.1 dB. Therefore, the Structure 1 with *Au*/*DNA*/*Au* achieves the convertible between two opposite modes. Figure 3a,c shows the electric field intensity distribution at the end of the Structure 1 for the ON and OFF modes. The distribution characteristics of the electric fields are directly related to the energy conduction properties of the electromagnetic wave along the DNA waveguide. For example, when the resistance is reduced, the ON mode is achieved in the DNA strip, the electric field intensity is mainly distributed in the output port of the waveguide, as shown in Fig. 3a. These electric field intensity distributions indicate that most of the electromagnetic energy is transmitted to the output port, as shown in Fig. 3a. However, for the OFF mode of the DNA strip, the electric field intensity is reduced, which indicates that most of the electromagnetic energy is blocked from reaching the output port, as shown in Fig. 3c. When the gold layers are replaced with silver layers, the Structure 1 with *Ag*/*DNA*/*Ag* is achieved, as shown in Fig. 3b,d. The Structure 1 with *Ag*/*DNA*/*Ag* shows similar electromagnetic wave energy conduction property. The distribution of electric field intensity at the output port can be modulated by changing the resistance of the DNA layer, as shown in Fig. 3b,d. Metal materials in three proposed Structures directly affect electric field strength and the ratio between two modes is set to be field enhancement factor (FEF):

5 $$FEF = \left| {\frac{{E_{ON} }}{{E_{OFF} }}} \right|$$

Here, $E_{ON}$ is the maximum electric field strength at the output port of the structure (in cross section of *xoz*) for the ON mode, while $E_{OFF}$ is the maximum electric field strength at the output port of the structure for the OFF mode. Based on the achieved results in Fig. 3a,c, the FEF reaches 2.18 ($E_{ON} \approx 1.27 \times 10^{8}$, $E_{OFF} \approx 5.83 \times 10^{7}$) for the proposed Structure 1 with *Au*/*DNA*/*Au*. For the Structure 1 with *Ag*/*DNA*/*Ag*, the return loss value is shown in Fig. 2, which is similar to the simulated results of the *Au*/*DNA*/*Au*. This is because the resistance and conductivity of the gold layers are similar to the silver layers. Therefore, the FEF reaches 2.07 ($E_{ON} \approx 1.24 \times 10^{8}$, $E_{OFF} \approx 5.98 \times 10^{7}$) for the proposed Structure 1 with *Ag*/*DNA*/*Ag*.

### Structure 2 with *Au*/*DNA*/*Dirac*, *Dirac*/*DNA*/*Au*, *Ag*/*DNA*/*Dirac*, or *Dirac*/*DNA*/*Ag*

**Figure 4:**
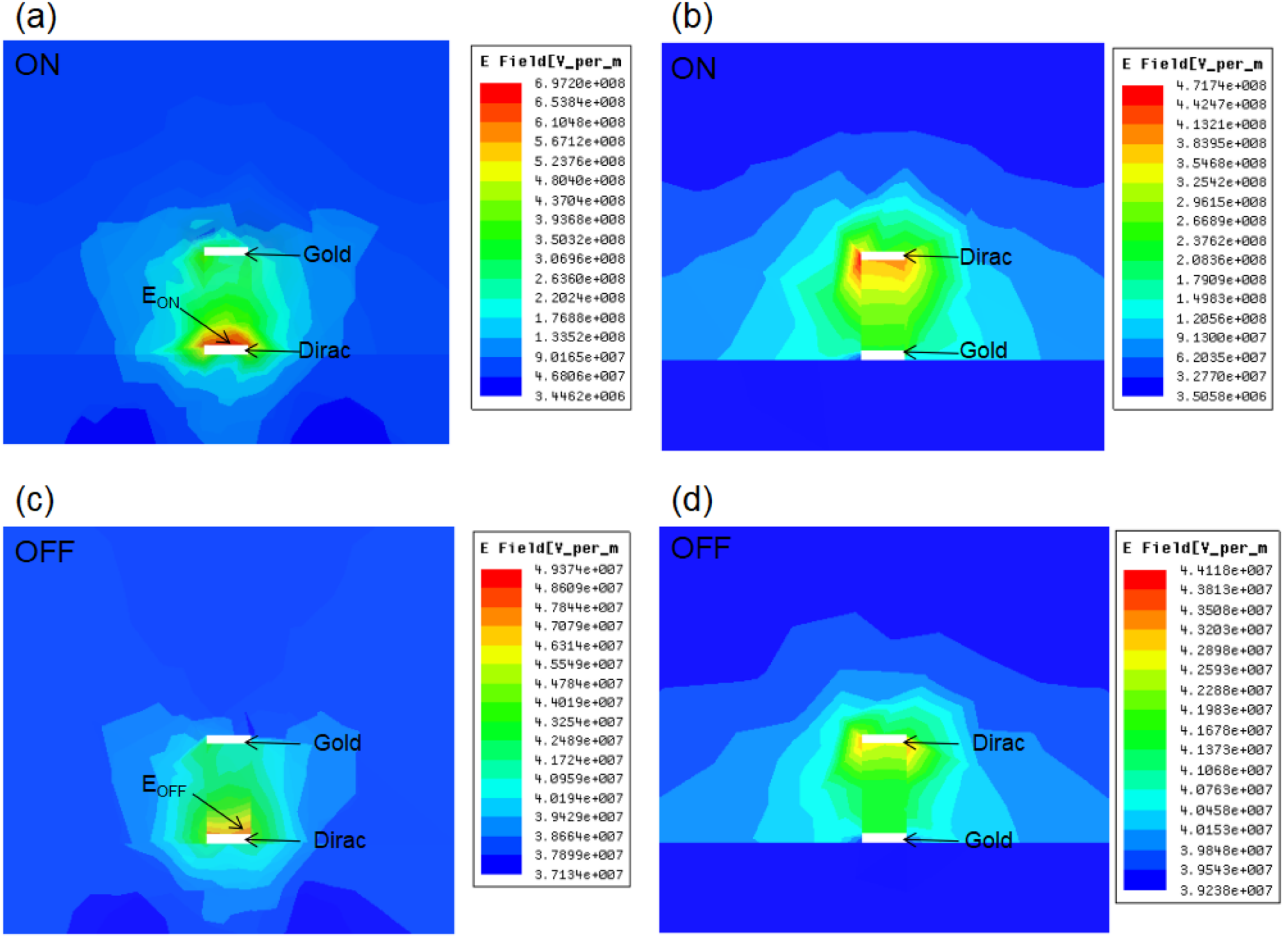
(**a**) The electric field intensity at the end of the waveguide for the ON mode of the Structure 2 with *Au*/*DNA*/*Dirac*. (**b**) The electric field intensity at the end of the waveguide for ON mode of the Structure 2 with *Dirac*/*DNA*/*Au*. (**c**) The electric field intensity at the end of the waveguide for OFF mode of the Structure 2 with *Au*/*DNA*/*Dirac*. (**d**) The electric field intensity at the end of the waveguide for OFF mode of the Structure 2 with *Dirac*/*DNA*/*Au*.

**Figure 5:**
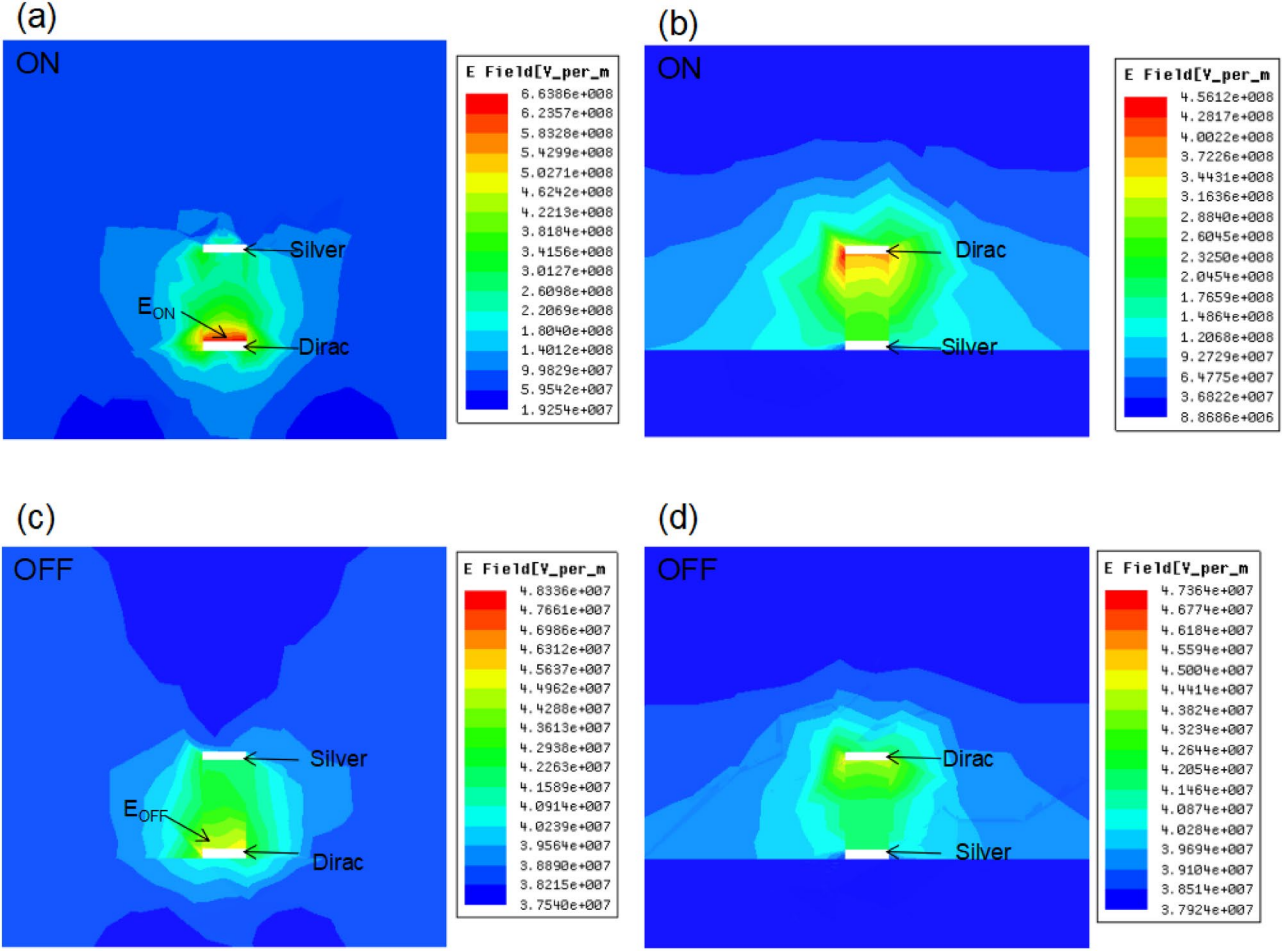
(**a**) The electric field intensity at the end of the waveguide for the ON mode of the Structure 2 with *Ag*/*DNA*/*Dirac*. (**b**) The electric field intensity at the end of the waveguide for ON mode of the Structure 2 with *Dirac*/*DNA*/*Ag*. (**c**) The electric field intensity at the end of the waveguide for OFF mode of the Structure 2 with *Ag*/*DNA*/*Dirac*. (**d**) The electric field intensity at the end of the waveguide for OFF mode of the Structure 2 with *Dirac*/*DNA*/*Ag*.

In order to enhance the propagation properties of this waveguide, Dirac semimetal is used in the proposed Structure 2. The Structure 2 contains four combinations: *Au*/*DNA*/*Dirac*, *Dirac*/*DNA*/*Au*, *Ag*/*DNA*/*Dirac*, and *Dirac*/*DNA*/*Ag*. Figure 4a,c shows the electric field intensity distribution at the output port of the Structure 2 with *Au*/*DNA*/*Dirac* for the ON and OFF modes. The electric field intensity distribution at the output port is higher than that of Structure 1 in Fig. 3a,b for the ON mode, as shown in Fig. 4a. When the DNA strip is switched to the OFF mode, the electric field intensity is drastically reduced, which is lower than that of both Structure 1 in Fig. 3c,d for the OFF mode, as shown in Fig. 4c. For the Structure 2 with *Dirac*/*DNA*/*Au*, the electric field intensity is also concentrated in the area next to the Dirac semimetal layer, which is also higher than that of Structure 1 in Fig. 3a,b for the ON mode, as shown in Fig. 4b. When the OFF mode is achieved, the electric field intensity is drastically reduced, as shown in Fig. 4d. When the metal layer is replaced with a Dirac layer, two structural design strategies (*Dirac*/*DNA*/*Ag* or *Ag*/*DNA*/*Dirac*) are achieved. Moreover, similar resonance behaviors are also achieved, as shown in Fig. 5. The simulation results show that although Structure 2 contains four different combinations of metal layers (*Au*/*DNA*/*Dirac*, *Dirac*/*DNA*/*Au*, *Ag*/*DNA*/*Dirac*, and *Dirac*/*DNA*/*Ag*), the performance of these structural strategies is indeed similar. In order to illustrate accurately the performance of Structure 2, Table 2 gives the statistical results of Figs. 4 and 5. Obviously, Structure 2 has higher electric field enhancement factors than that of the structure 1 (*Au*/*DNA*/*Au* or *Ag*/*DNA*/*Ag*), which indicates that the Structure 2 has a better waveguide effect than the Structure 1. This is because that a Dirac semimetal layer with higher conductivity than the gold and silver layers is applied for the proposed Structure 2. Four FEF values are achieved by the Structure 2: 14.11, 10.70, 13.75, and 9.62, as shown in Table 2. The achieved FEF values based on the *Au*/*DNA*/*Dirac* and *Dirac*/*DNA*/*Au* are generally higher than that based on the *Ag*/*DNA*/*Dirac* and *Dirac*/*DNA*/*Ag*, but the difference is not large.

**Table 2:** FEF values of the Structure 2 with *Au*/*DNA*/*Dirac*, *Dirac*/*DNA*/*Au*, *Dirac*/*DNA*/*Ag*, and *Ag*/*DNA*/*Dirac.*

Electric field intensity	*Au*/*DNA*/*Dirac*	*Dirac*/*DNA*/*Au*	*Ag*/*DNA*/*Dirac*	*Dirac*/*DNA*/*Ag*
$E_{ON}$	6.97*10^8^	4.72*10^8^	6.64*10^8^	4.56*10^8^
$E_{OFF}$	4.94*10^7^	4.41*10^7^	4.83*10^7^	4.74*10^7^
FEF	14.11	10.70	13.75	9.62

### Structure 3

**Figure 6:**
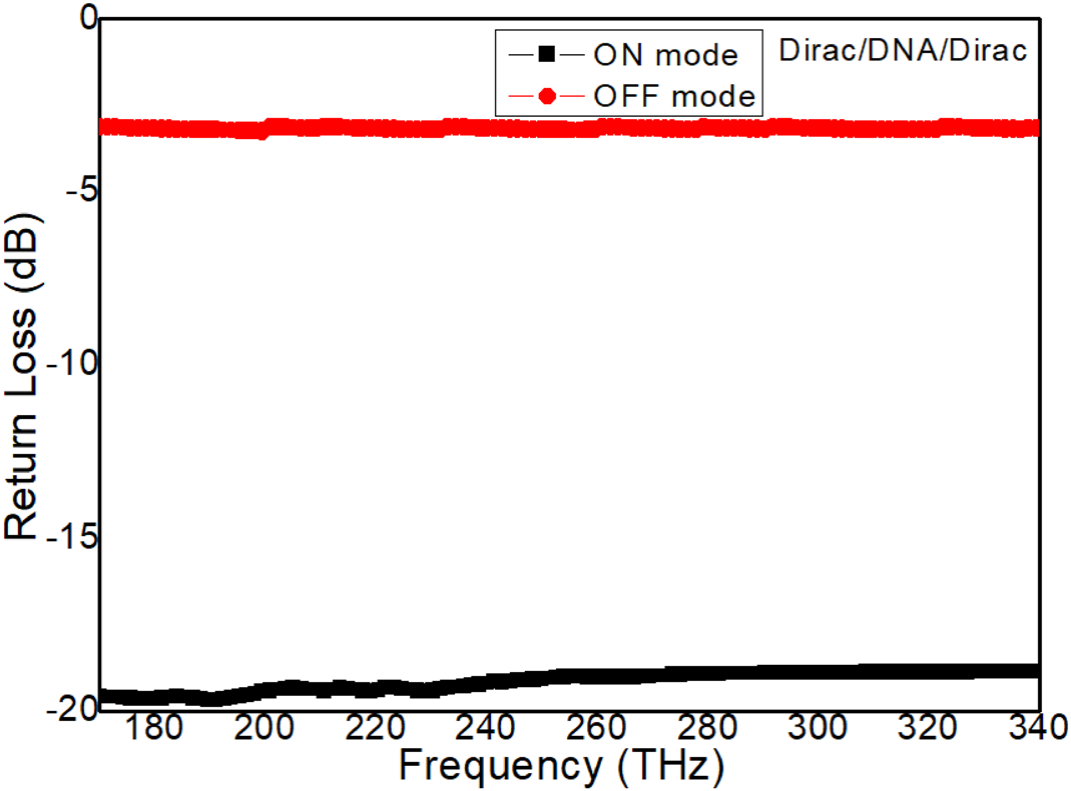
The return loss value of the *Dirac*/*DNA*/*Dirac* metamaterial waveguide for the ON and OFF modes.

**Figure 7:**
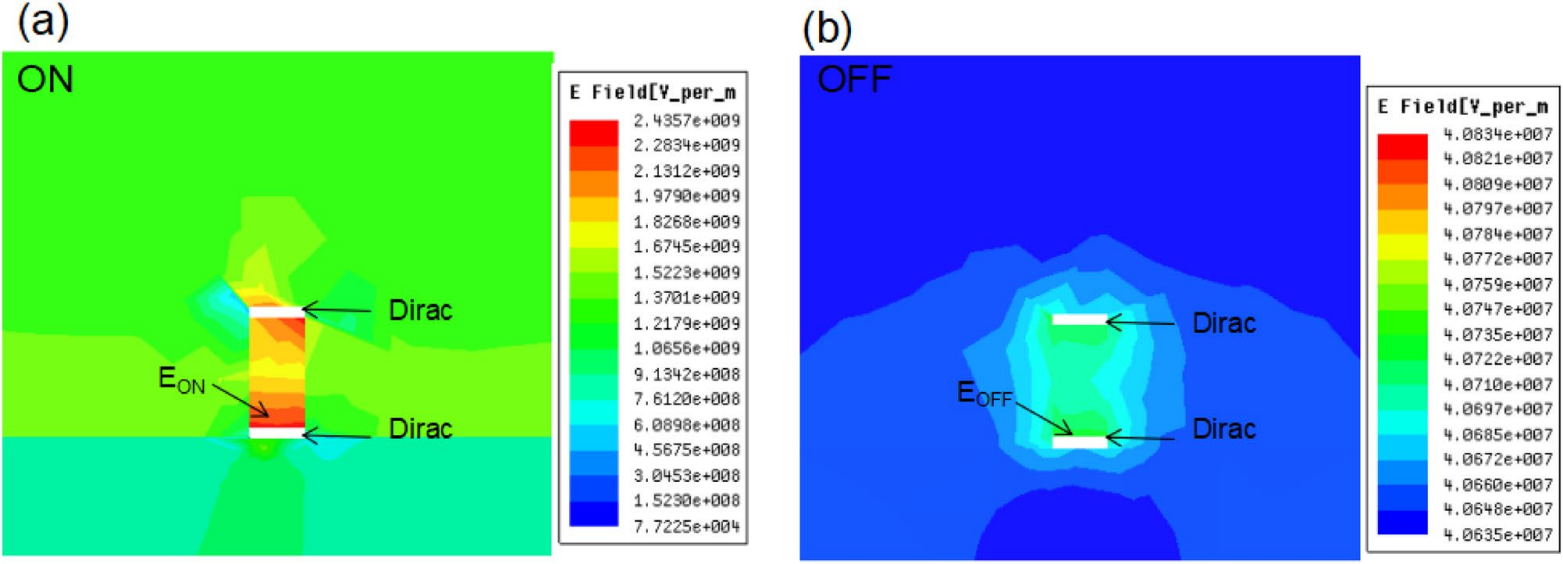
(**a**) The electric field intensity at the end of the waveguide for the ON mode of the Structure 2 with *Dirac*/*DNA*/*Dirac*. (**c**) The electric field intensity at the end of the waveguide for OFF mode of the Structure 2 with *Dirac*/*DNA*/*Dirac*.

To reveal the role of the Dirac semimetal, two Dirac semimetal layers are applied in the Structure 3. Figure 6 shows the return loss value of the Structure 3 with *Dirac*/*DNA*/*Dirac* for the ON and OFF modes. The Structure 3 under the ON mode shows a return loss less than that of both Structures 1 and 2, as shown in Fig. 6. Moreover, the calculated return loss for the ON mode exhibits a low amplitude oscillation in the low frequency band, and the amplitude decreases as the frequency increases. Figure 7 gives the electric field distributions for the ON and OFF modes of the Structure 3. It is revealed that the electric field strength at the output port of the Structure 3 for the ON mode is very higher than that of both Structures 1 and 2, as shown in Fig. 7a, while it is lower than that of both Structures 1 and 2 for the OFF mode, as shown in Fig. 7b. These results indicate that the Structure 3 can reveal a higher waveguide effect than the Structures 1 and 2, which also implies that the Dirac semimetal is suitable for use for waveguides in this frequency band. The influence of structural parameters *t*_*1*_ and *w* on the properties of the Structure 3 is simulated (the Fermi energy is 0). On the one hand, structural parameter *t*_*1*_ is set to be 10 nm, 12 nm, and 14 nm (Other structural parameters and simulation conditions remain unchanged). Three electric field enhancement factor are obtained: 59.80, 62.43, and 65.37. On the other hand, structural parameter *w* is set to be 80 nm, 85 nm, and 90 nm. Three electric field enhancement factor are also achieved: 59.80, 60.98, and 62.21. The simulation results show that structural parameter *t*_*1*_ has a higher impact on the performance of structure 3 than structural parameter *w*. This is because the increase in thickness can significantly reduce the resistance of the Dirac semi-metal layer.

**Figure 8:**
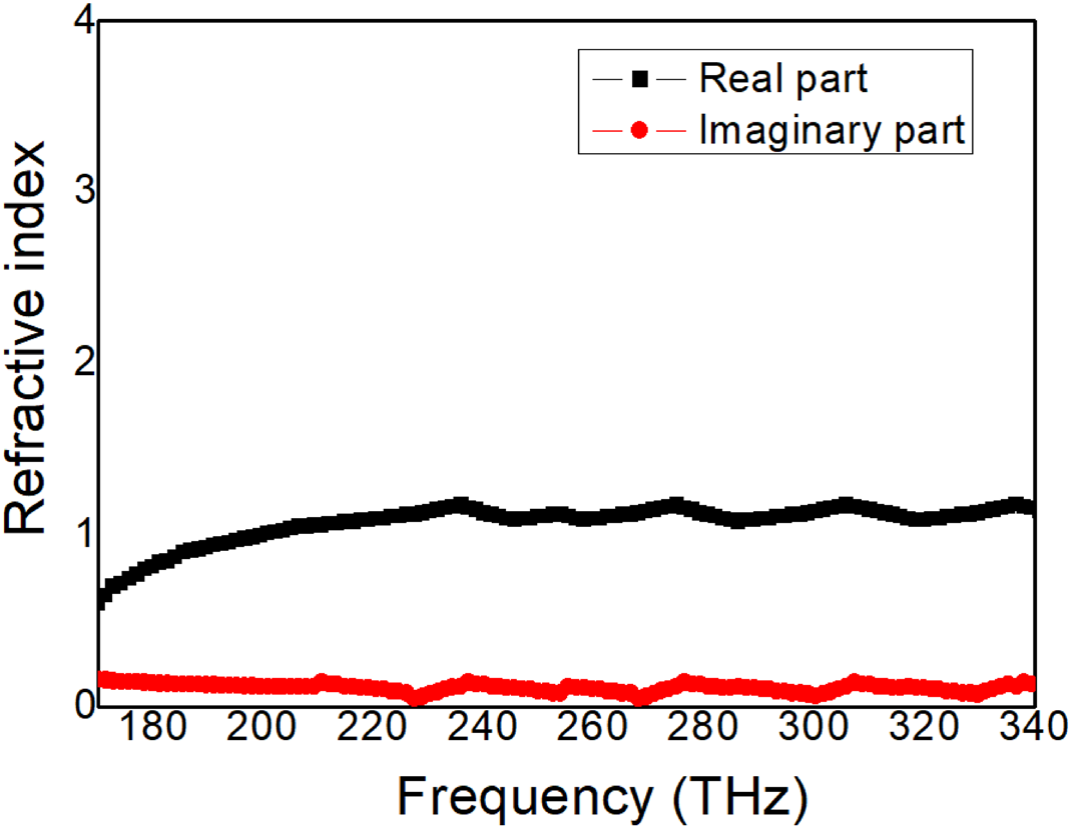
The real and imaginary parts of the effective refractive index of the proposed Structure 3.

The effective refractive index is an important parameter describing the resonance behavior of metamaterial devices^40–42^. The effective refractive index of Structure 3 is extracted, as shown in Fig. 8. The real part of the refractive index has a small change in the target frequency band, close to 1, which shows that the metamaterial waveguide is close to impedance matching. At the same time, the imaginary part of the refractive index is close to 0, which indicates that the absorption loss of the metamaterial waveguide is relatively small.

The convertibility of the Structure 3 between the ON and OFF modes is shown in Figs. 6 and 7. Furthermore, both of the ON and OFF modes can also be continuously modulated based on external conditions. This is due to the Dirac semimetal is sensitive to electromagnetic conditions. The real and imaginary parts of the conductivity can both controlled through changing the Fermi energy^43,44^. Moreover, the permittivity of the Dirac semimetal layers is also influenced by the Fermi energy according to the Eq. (4). Therefore, it is feasible to control the ON and OFF modes by changing Fermi energy. Table 3 shows the FEF values of the Structure 3 under different Fermi energy conditions. It is shown that the electric field strength for the ON mode is enhanced with the Fermi energy increasing, while the electric field strength for the OFF mode almost unchanged. Furthermore, the influence of the external magnetic field can’t be ignored. The resistance of the Dirac semimetal layers can be reduced by the external magnetic field based on the negative magnetoresistance effect^45,46^:

6 $$\triangle\sigma (B) \cong \alpha \frac{{ - e^{2} }}{{2\pi^{2} \hbar }}\left[ {\psi \left( {\frac{1}{2} + \frac{{B_{\varphi } }}{B}} \right) - \ln \left( {\frac{{B_{\varphi } }}{B}} \right)} \right] + c B^{2}$$

**Table 3:** FEF values of the Structure 3 with *Dirac*/*DNA*/*Dirac* with different Fermi energy.

Electric field intensity	0 meV	20 meV	40 meV	60 meV
$E_{ON}$	2.44*10^9^	2.96*10^9^	3.58*10^9^	4.23*10^9^
$E_{OFF}$	4.08*10^7^	4.25*10^7^	4.41*10^7^	4.60*10^7^
FEF	59.80	69.65	81.18	91.96

In the above Eq. (6), *B* is the magnetic field intensity, $\alpha$ means the weak localizaton. $B_{\varphi } { = }\frac{h}{{4eL_{\varphi }^{2} }}$ is set to be the characteristic field. The dephasing length is revealed to be $L_{\varphi }$. $\psi (x)$ is set to be the digamma function. As the magnetic field strength increases, the resistance of Dirac semimetal is reduced due to the negative magnetoresistance effect, which leads to the surface current of the Dirac semimetal layers enhance. Table 4 shows the FEF values of the Structure 3 under different magnetic field strength conditions. Similarly, the electric field strength for the ON mode is also enhanced base on the negative magnetoresistance effect, while it is almost unchanged for the OFF mode with the magnetic field strength increasing. These simulated results indicate that both modes can be continuously modulated through changing external electromagnetic conditions, as shown in Tables 3 and 4.

**Table 4:** FEF values of the Structure 3 with *Dirac*/*DNA*/*Dirac* with different magnetic field strength.

Electric field intensity	0.0 T	0.3 T	0.6 T	0.9 T
$E_{ON}$	2.44*10^9^	2.51*10^9^	2.62*10^9^	2.75*10^9^
$E_{OFF}$	4.08*10^7^	4.12*10^7^	4.26*10^7^	4.39*10^7^
FEF	59.80	60.90	61.50	62.64

## Conclusion

In this paper, a tunable metamaterial based on DNA strip is designed in 170-340THz range. Two opposite modes (ON and OFF) can be achieved based on the low and high resistance states of the DNA strip. Three structural schemes are proposed and validated: Structure 1 (*Au*/*DNA*/*Au* or *Ag*/*DNA*/*Ag*), Structure 2 (*Au*/*DNA*/*Dirac*, *Dirac*/*DNA*/*Au*, *Ag*/*DNA*/*Dirac*, or *Dirac*/*DNA*/*Ag*), Structure 3 (*Dirac*/*DNA*/*Dirac*). The Structure 3 can achieved a higher FEF value than that of the Structures 1 and 2. Based on the tunability of the Dirac semimetal, the FEF value of Structure 3 can be modulated by varying the Fermi energy and the magnetic field strength. The proposed structure reveals a switchable feature based on the resistance characteristics of DNA strips, which can be applied for optical memristor or optical gate.
